# *Tropheryma whipplei* escapes LAPosome and modulates macrophage response in a xenophagy-dependent manner

**DOI:** 10.1080/27694127.2025.2475527

**Published:** 2025-03-11

**Authors:** Emilie Reyne, Jeffrey Arrindell, Eloïne Bestion, Soraya Mezouar, Benoit Desnues

**Affiliations:** a Aix-Marseille Univ, MEPHI, Marseille, France; b IHU-Méditerranée Infection, Marseille, France; c Genoscience Pharma, 13006 Marseille, France; d Aix-Marseille Univ, ADES, CNRS, EFS, Marseille, France; e lead contact

**Keywords:** Autophagy, Immune escape, LC3-associated phagocytosis (LAP), Macrophage, *Tropheryma whipplei*, Whipple’s disease

## Abstract

*Tropheryma whipplei*, the agent of Whipple’s disease, is an intracellular pathogen that replicates in macrophages. The phagocytic and cellular processes leading to the formation of *T. whipplei* replicative vacuole remain poorly understood. Macrophage microbicidal activity is largely related to macro/autophagy which is also essential for cell homeostasis. Here, we show that *T. whipplei* uptake by macrophages involved LC3-associated phagocytosis (LAP). Bacteria then escaped into the cytosol from where they were recaptured by xenophagy. We also demonstrate that *T. whipplei* blocked the autophagic flux to build its replicative compartment. Inhibition of LAP resulted in the decrease of interleukin (IL)-10 secretion and the restoration of the autophagy flux, suggesting that modulation of autophagy during infection alters immune response and promote persistence. Our results provide new insight in the intracellular fate of the bacteria during macrophage infection and suggest the possible involvement of previously unknown virulence factors in *T. whipplei* infection.

## Introduction

Macroautophagy (hereafter called autophagy) guarantees cellular homeostasis by capturing and degrading intracellular damaged organelles, long-lived or misfolded proteins and aggregates but also invading pathogens free in the cytosol (referred in this last case to xenophagy). All these cargoes are targeted by different receptor-proteins (including SQSTM1/p62 [sequestosome 1] or NDP52 [nuclear dot protein 52]) that permit the recruitment and the elongation of the autophagosome (whose protein signature is LC3 [Microtubule-associated proteins 1A/1B light chain 3B]) and have the lysosomal compartment as destination. Because of its important crosstalk with the inflammasome and apoptosis, autophagy is as well central to the immune response [1,2], particularly in phagocytic and antigen-presenting cells such as macrophages and dendritic cells [3]. It has also been described that autophagy can be associated with phagocytosis, a process known as LC3-associated phagocytosis (LAP), resulting in the formation of a LAPosome, a single membrane vesicle decorated with LC3. LAP involves specific effectors, including RUBCN (RUN and cysteine rich domain containing BECN1/BECLIN1-interacting protein), which then recruits NADPH oxidase [4]. Consequently, it was shown that LAP is required for killing of *Listeria monocytogenes* by macrophages and contribute to immunity of mice, whereas canonical autophagy is dispensable [5]. In addition, deficiency in autophagy genes and NADPH oxidase impairs host defense against *Salmonella enterica* serovar Typhimurium, although full control of *S. Typhimurium* replication requires other mechanisms than LAP and ROS (reactive oxygen species) production [6].

Many bacteria are specialized in thwarting their elimination by interfering with the autophagic machinery [7,8]. This is particularly true for *Mycobacterium tuberculosis* which neither LAP nor xenophagy can eliminate. The secreted protein CspA (a LytR-CpsA-Psr [LCP] domain-containing protein) protects the bacterium from lysosomal clearance and death by inhibiting LAP through interference with NADPH oxidase subunit recruitment and subsequent production of ROS [9]. In the meantime, *M. tuberculosis* secretes across its type VII secretion system ESAT6 (early secreted antigenic target of 6 kDa, also known as EsxA), a major virulence factor that induces phagosomal membrane rupture, allowing mycobacteria to gain access to the cytosol [10,11]. *M. tuberculosis* gets then ubiquitinated and recognized by the cytosolic autophagy receptors SQSTM1/p62 and NDP52 which initiates autophagosome formation. The ubiquitin ligase Parkin 2 and Smurf1 are of critical importance for the ubiquitination of the bacteria [12].

*Tropheryma whipplei* is the causative agent of the Whipple’s disease, a rare, chronic, and systemic disease characterized by joint involvement, digestive disorders, and sometimes neurological implications [13]. Infiltration of foamy macrophages filled with bacteria is the hallmark histological lesion. *T. whipplei* infects macrophages [14] and replicates inside an acidic phagosome [15] expressing lysosome-associated membrane glycoprotein 1 (LAMP1) but lacking cathepsin D, suggesting a defect in fusion of the late phagosome to the lysosome [16]. This would result from the high secretion of interleukin (IL)-16 during infection [17,18]. Within the macrophage, *T. whipplei* induces an alternative or M2 polarization [19] which remains atypical, with a weak tumor necrosis factor (TNF) response [20], associated with IL-1β secretion, induction of apoptosis [21], and type I interferon (IFN) [22].

The phagocytic and cellular processes leading to *T. whipplei* replicative vacuole remain relatively unknown. Here we demonstrate that, in macrophages, *T. whipplei* is internalized by LAP, but this is not sufficient for bacterial clearance. Instead, *T. whipplei* escapes into the cytosol and is further recaptured by xenophagy without being eliminated, as the bacterium hijacks the autophagosome to form its own replicative compartment. We also observe a late-stage blockade of autophagic flux that lasts over time and is concomitant with RUBCN over-expression, suggesting that LAP-mediated bacterial uptake favors persistent infection. Our findings provide new insight into the intracellular history of *T. whipplei* during macrophage infection.

## Results

### *Uptake of* T. whipplei *by macrophages involves LAP*

**Figure 1.**
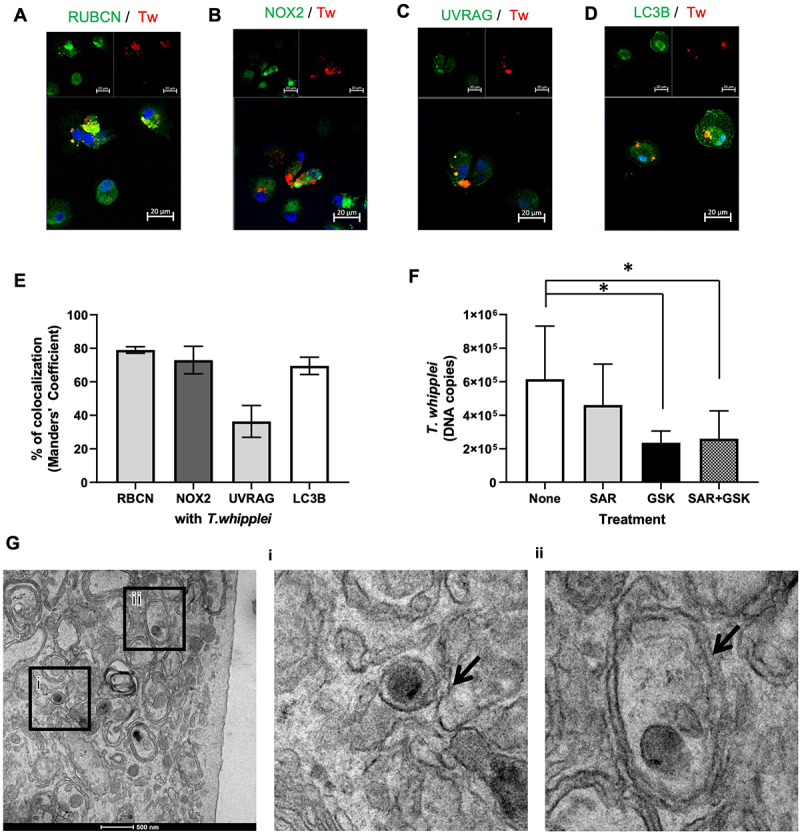
*T. whipplei* induces LC3-associated phagocytosis in macrophages. MDMs were infected with *T. whipplei* (50 bacteria per cell) for 2 hours, fixed, then stained with an anti-*T. whipplei* antibody in red and antibodies directed against RUBCN (A), NOX2 (B), UVRAG (C), or LC3B (D) in green. Nuclei were stained with DAPI (blue). The images were visualized by confocal fluorescence microscopy. Scale bar: 20 µm. (E) Colocalization between *T. whipplei* and the proteins of interest was expressed as Manders coefficient. (F) MDMs were pre-treated with 10 µM of SAR405 and/or 25 µM of GSK2795039 for 4 hours before been infected for 2 hours with *T. whipplei* (50 bacteria per cell), washed to remove free bacteria and then lysed. Bacterial DNA copies was determined by qPCR. The experiment was performed using three different donors (N = 3), and the values represent the mean ± standard error of the mean. *p<0.05, by two-way ANOVA. (G) MDMs were infected with *T. whipplei* (50 bacteria per cell) for 2 hours, then were fixed and analyzed by transmission electron microscopy; arrow in (i) shows bacteria inside a simple membrane structure and arrow in (ii) shows bacteria inside a double-membrane structure. Scale bar: 500 nm. The experiments were performed on three different donors (N = 3) and representative results are shown.

In a first set of experiments, we wondered whether autophagy machinery was involved during *T. whipplei* infection of monocyte-derived macrophages (MDMs, bacterium-to-cell ratio of 50:1). We found that 2 hours after infection, almost 80% of bacteria were associated with RUBCN as revealed by colocalization experiments (Figure 1A and 1E). In addition, *T. whipplei* also colocalized at 70% with NOX2 (Figure 1B and 1E), 40% UVRAG (Figure 1C and 1E) and 80% with LC3B (Figure 1D and 1E) suggesting that macrophages could internalize bacteria through LAP.

To test whether LAP was conducive to bacterial internalization in macrophages, cells were pre-treated 4 hours prior infection with SAR405, a PIK3C3/Vps34 kinase inhibitor and/or with the NOX2-specific inhibitor GSK2795039. In the presence of SAR405, RUBCN did not seem to colocalize with *T. whipplei* (Figure S1A), while GSK2795039 seemed to have no effect on NOX2 recruitment to *T. whipplei* phagosome (Figure S1B). Interestingly, we found that treatment of macrophages with GSK2795039 alone or with GSK2795039 concomitantly with SAR405 resulted in a significant but not complete reduction of the number of internalized bacteria (Figure 1F). Likewise, as a certain number of bacteria may remain associated to the cell surface despite extensive washing after the 2-hour infection, we treated the cells with gentamycin for 2 additional hours to ensure that no live bacteria are further internalized. However, we did not find significant difference between gentamycin-treated and untreated cells (Figure S1C), suggesting that LAP is involved, but not mandatory for *T. whipplei* internalization by macrophages. Finally, closer examination of the bacteria-containing vacuoles by transmission electron microscopy (TEM) revealed that most bacteria were found in single-membrane vacuoles (Figure 1Gi) while some of them were observed in double-membrane compartments (Figure 1Gii). Further vacuole quantification by TEM on 160 vacuoles from 50 different fields showed that approximately 65% of them were single-membrane while 35% were double-membrane, strengthening the fact that autophagic processes are activated during *T. whipplei* uptake.

Altogether, these data indicate that macrophages internalize *T. whipplei* in a mechanism which at least in part depends on LAP.

### *Escaped cytosolic* T. whipplei *are recaptured by xenophagy*

**Figure 2.**
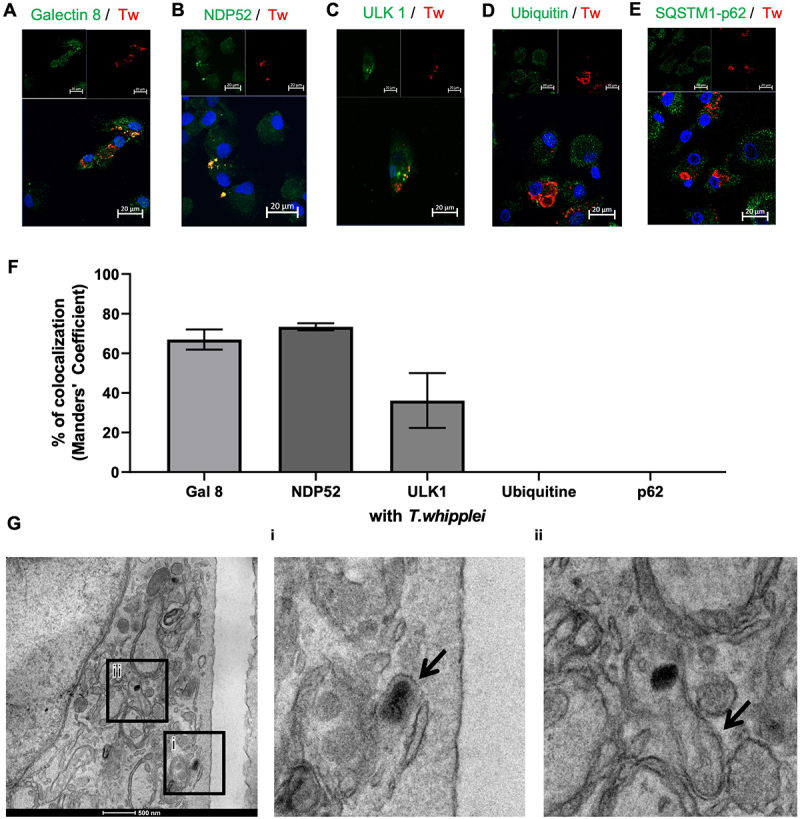
*T. whipplei* escapes before being recaptured by xenophagy. MDMs were infected 2 hours with live or paraformaldehyde-inactivated *T. whipplei* (50 bacteria per cell), fixed then stained with an anti-*T. whipplei* antibody in red, and antibodies directed against Galectin 8 (A), or NDP52 (B), ULK1 (C), Ubiquitin (D) or SQSTM1/p62 (E) in green. Nuclei were stained with DAPI (blue). The images were visualized by confocal fluorescence microscopy. Scale bar: 20 µm. Colocalization between *T. whipplei* and the proteins of interest was expressed as Manders coefficient (F). (G) MDMs were infected with *T. whipplei* (50 bacteria per cell) for 2 hours, then fixed and analyzed by transmission electron microscopy; arrow in (i) shows bacteria free in the cytosol near a simple membrane structure and arrow in (ii) shows bacteria near a double-structure membrane like autophagore elongation. Scale bar: 500 nm. The experiments were performed in triplicates (N = 3); representative results are shown.

The fact that some bacteria were seen in double-membrane vacuoles strongly suggests that *T. whipplei* escapes the phagosome and is further recaptured by xenophagy. To test this hypothesis, we first focused on the cytosolic autophagy receptor NDP52 and the cytosolic lectin galectin 8. We found that *T. whipplei* colocalized at 70% with galectin 8 (Figure 2A and 2F) and at 75% with NDP52 (Figure 2B and 2F). This was dependent on bacterial viability since inactivation of bacteria with paraformaldehyde prevented bacterial interaction with NDP52 (Figure S1D) strongly suggesting that some bacteria were able to actively escape the phagosomal vacuole. Interestingly, galectin 8 and NDP52 may further activate autophagy since around 40% of bacteria colocalized with ULK1 (Figure 2C and 2F), which is required for initiation of canonical autophagy but not for LAP. Surprisingly, ubiquitin and SQSTM1/p62 did not colocalize with the bacteria (Figure 2D, E and F), suggesting the ubiquitin-mediated xenophagy was deficient. Similarly, components of the Endosomal Sorting Complexes Required for Transport (ESCRT), which also require ubiquitin binding [23] were not recruited to *T. whipplei* since TSG101 (ESCRT-I complex), CHMP4A (ESCRT-II complex) and VPS4, which triggers membrane scission and ESCRT-III disassembly, did not colocalized with *T. whipplei* (Figure S2B). These data were further confirmed by transmission electron microscopy which revealed free bacteria in the cytosol but also bacteria close to a double membrane undergoing elongation, characteristic of canonical autophagy/xenophagy (Figure 2G).

Taken together, these observations suggest that *T. whipplei* escape the LAPosome by an active mechanism and that escaped bacteria are then recaptured by xenophagy.

### T. whipplei *infection blocks the autophagic flux at late stage*

**Figure 3.**
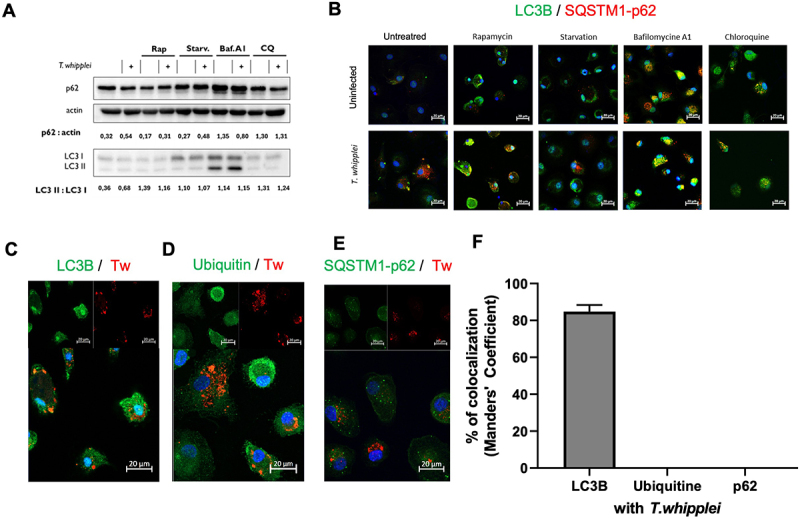
*T. whipplei* infection blocks autophagic flux in macrophages. MDMs were pre-treated 4 hours with 500 nM rapamycin, or starved for 16 hours, or treated with 200 nM bafilomycin A1, or 60 µM chloroquine, before infection with *T. whipplei* (50 bacteria per cell) for 24 hours. (A) Cells were then washed and lysed. Whole-cell lysates were then analyzed by western blot against the indicated proteins. (B) MDMs were washed, fixed and then stained with anti-LC3B in green, anti-SQSTM1-p62 in red and DAPI to identify nuclei in blue. Yellow puncta show cargoes. (C-E) MDMs were infected or not by *T. whipplei* (50 bacteria per cell) for 4 hours, washed and incubated for 24 hours, washed, fixed and stained with an anti-*T. whipplei* antibody in red, an anti-LC3B (C), Ubiquitin (D) or SQSMT1/p62 (E) in green. Nuclei were stained with DAPI (blue). (F) Colocalization between *T. whipplei* and proteins of interest was expressed as Manders coefficient. The images were visualized by confocal fluorescence microscopy. Scale bar: 20 µm. The experiments were performed in triplicates (N = 3); representative results are shown.

We next wondered whether infection would impact the autophagic flux. Macrophages were infected for 24 hours, with or without modulating drugs or treatment, and the autophagic flux was monitored by western blot by analyzing the levels of SQSTM1/p62 and LC3-II:LC3-I ratio. We found that *T. whipplei* seems to induce the retention of SQSTM1/p62 and LC3-II, as well a further decrease in LC3-I expression compared to control, resulting in the global increase of the LC3-II:LC3-I ratio (Figure 3A and S3B), although a certain amount of heterogeneity across samples was observed (Figure S3A and S3B). Interestingly, marked accumulation of LC3-II and SQSTM1/p62 was observed with the late-stage inhibitors of autophagy bafilomycin A1 and with chloroquine (Figure 3A, S3A and S3B) in both infected and uninfected cells. The induction of canonical autophagy by rapamycin lead to a consumption SQSTM1/p62 and a decrease of LC3-II compared to untreated control cells, this effect being slightly diminished in the presence of *T. whipplei* (Figure 3A, S3A and S3B), suggesting that the blockade of autophagy by *T. whipplei* could not be reversed by induction of canonical autophagy.

These results were further confirmed by confocal microscopy which showed that LC3/p62 cargoes accumulated in infected cells (Figure 3B). As expected, we found that in the presence of the autophagy inhibitors bafilomycin A1 and chloroquine, LC3B formed puncta with SQSTM1/p62 (Figure 3B). Although *T. whipplei* still colocalized at 85% with LC3B after 24h (Figure 3C and 3F), bacteria were not found in SQSTM1/p62-positive and ubiquitinated compartments (Figure 3D, E and F).

Overall, these results suggest that *T. whipplei* blocks the autophagic flux at late stage, i.e. maturation/fusion with the lysosome, thereby favoring bacterial maintenance.

### T. whipplei *hijacks autophagy to build up its replicative compartment*

We next focused on the *T. whipplei* replicative compartment. Macrophages were infected for 4 hours, washed to remove extracellular bacteria, and incubated for 24 hours before investigation.

**Figure 4.**
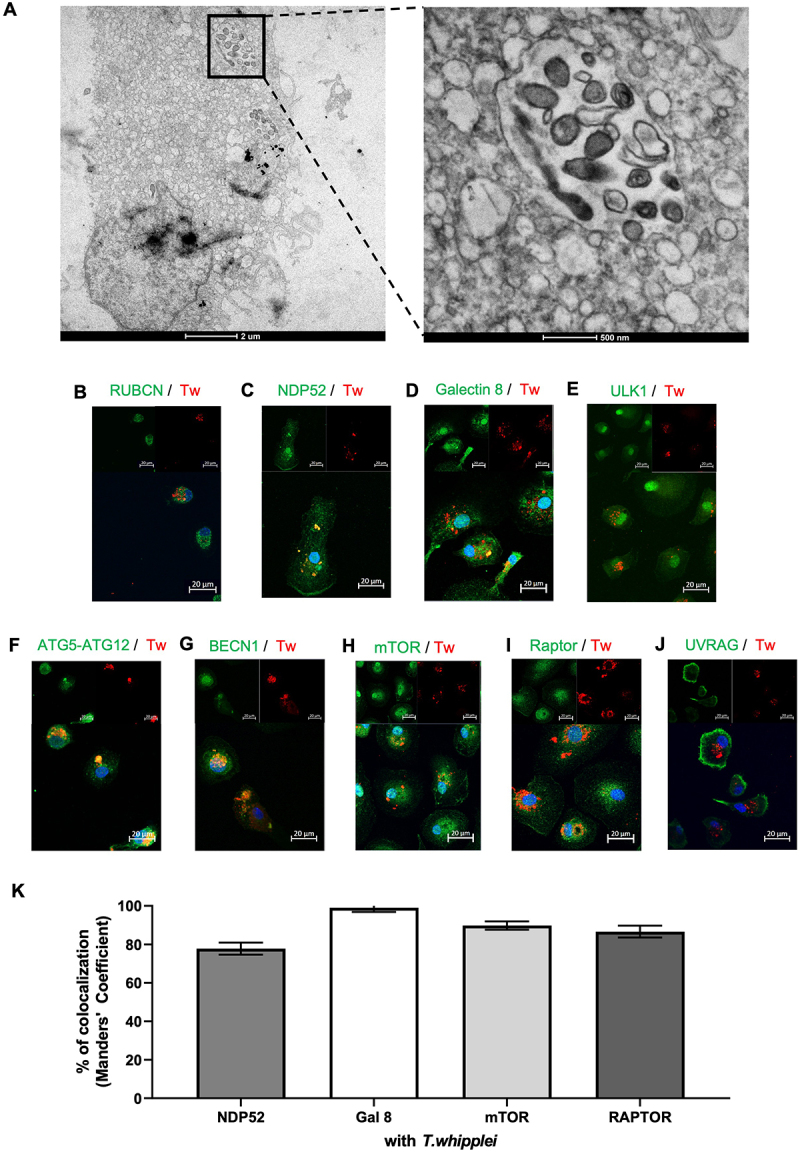
*T. whipplei* hijacks autophagy to build up its replicative compartment. MDMs were infected with *T. whipplei* (50 bacteria per cell) for 4 hours, washed and incubated for 24 hours, then fixed and analyzed by transmission electron microscopy (A) or by confocal immunofluorescence after staining with anti-*T. whipplei* antibody in red, and antibodies directed against RUBCN (B), NDP52 (C), Galectin 8 (D), ULK1 (E), an ATG5-ATG12 (F), BECN1 (G), m-TOR (H), Raptor (I), or UVRAG (J) in green. (K) Colocalization between *T. whipplei* and the proteins of interest was expressed as Manders coefficient. Nuclei were stained with DAPI (blue). Scale bar: 20 µm. The experiments were performed on three different donors (N = 3); representative results are shown.

The presence of large, double-walled, electron-dense vacuoles that can be identified as phagosomes, was observed containing bacteria (Figure 4A). *T. whipplei*-containing vacuoles were no longer associated with RUBCN (Figure 4B) while bacteria still colocalized at 75% with NDP52 (Figure 4C and 4K) and at 90% with galectin 8 (Figure 4D and 4K), but not with members of the ESCRT system (Figure S2C). In addition, *T. whipplei*-containing vacuoles did not express ULK1 (Figure 4E), ubiquitin (Fig 3D) nor SQSTM1/p62 (Figure 3E) but acquired autophagic effectors such as ATG5-12 (Figure 4F), BECN1 (Figure 4G) and still expressed LC3B (Figure 3C), which are effectors of the phagophore initiation, nucleation, and elongation stages, respectively. We also observed colocalization of *T. whipplei* with elements of the mTOR complex 1 as 90% of bacteria colocalized with mTOR (Figure 4H and 4K) and 85% with Raptor (Figure 4I and 4K). Finally, UVRAG, which is necessary for the formation of the PI3K complex II for fusion of the cargoes to the lysosome, was not recruited to the vacuole but was segregated at the cell periphery (Figure 4J).

Overall, these findings indicate that *T. whipplei* resides in an autophagic vacuole with a maturation defect.

### *Disabling LAP of* T. whipplei *alters the immune response of macrophages*

**Figure 5.**
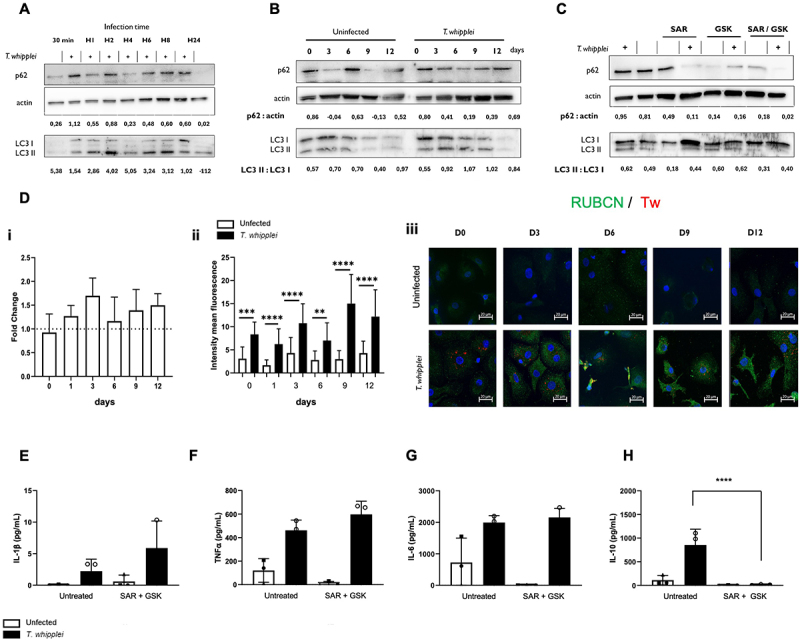
LAP induction promotes autophagy blockade and alters the immune response during *T. whipplei* infection. (A) MDMs were infected with *T. whipplei* (50 bacteria per cell) for the indicated times, then washed and lysed. Whole-cell lysates were then analyzed by western blot against the indicated proteins. (B) MDMs were infected with *T. whipplei* (50 bacteria per cell) 4 hours, washed to remove free bacteria and incubated for 12 days (0 corresponds to 4h infection). Every 3 days, cells were washed and lysed with RIPA buffer. Whole-cell lysates were then analyzed by western blot against the indicated proteins. (C) MDMs were pre-treated with 10 µM of SAR405, 25 µM of GSK2795039 or both for 4 hours and infected with *T. whipplei* (50 bacteria per cell) for 2 hours, Cells were washed and lysed with RIPA buffer. Whole-cell lysates were then analyzed by western blot against the indicated proteins. (D) MDMs from different donors (N=3) were infected with *T. whipplei* (50 bacteria per cell) for 4 hours, washed and incubated for 12 days. Gene expression of *RUBCN* was monitored every 3 days (day 0 corresponds to 4 hours infection) by qRT-PCR and expressed as fold change after normalization to the *ACTB* endogenous control (i) or at the translational level by measuring mean fluorescence intensity of RUBCN stained cells by confocal microscopy (ii). **p<0.01, ***p<0.001, *****<0.0001 by one-way ANOVA. (iii) Representative immunofluorescences are shown with anti-*T. whipplei* in red, anti-RUBCN in green and DAPI in blue. (E-H) MDMs were pre-treated with both 10 µM of SAR405 and 25 µM of GSK2795039 4 hours before infection with *T. whipplei* (50 bacteria per cell). After 24 hours, supernatants were collected and levels of IL-1β (E), TNF (F), IL-6 (G) and IL-10 (H) were assessed by ELISA. The experiment was performed using different donors (N = 3), representative results are shown for western-blots, and the values represent the mean ± standard error. ****p<0.0001 by two-way ANOVA.

Finally, we evaluated the course of *T. whipplei*-induced blockade of the autophagic flux in macrophages over time. The retention of SQSTM1/p62 with LC3 was visible as early as 30 minutes and became firmly established after 8 hours of infection (Figure 5A). We next infected macrophages for 4 hours (day 0), washed them to remove free bacteria and incubated them over 12 days. Results showed *T. whipplei* infection induced a greater and prolonged retention of SQSTM1/p62 compared to uninfected conditions which protein expression varied with time (Figure 5B, Figure S3C). Similar observations were made for LC3-I and LC3-II (Figure 5B). Of note, it was difficult to monitor autophagic flux after 9 days, probably because maintaining the cells in the same culture medium for such a time induces starvation, a known inducer of autophagy [24]. These results suggest that *T. whipplei* modulates autophagy, probably through an early blockade of the autophagic flux.

Finally, we wondered whether LAP promotion and blockade of the autophagic flux were linked. Interestingly, we found that LAP inhibition reduced the accumulation of SQSTM1/p62 and LC3-II during infection but increased the portion of LC3-I (Figure 5C), suggesting that *T. whipplei* uptake by LAP contributes to the blockade of the autophagic flux. This observation was associated with a slight but increased expression of *RUBCN* transcript over the 12 days of incubation (Figure 5Di), which resulted in a significantly increased expression of RUBCN at the protein level in infected *versus* uninfected cells (Figure 5Dii and 5Diii) over time. This finding strongly suggests that RUBCN plays a role in the persistence of the autophagic flux blockade.

LAP is known to promote immune tolerance in myeloid cells by inducing an anti-inflammatory profile [25]. Thus, we investigated whether LAP induction could alter the cytokine response to *T. whipplei* infection. LAP inhibition did not affect proinflammatory cytokine secretion as the levels of IL-1β, TNF and IL-6 were not affected 24 hours post-infection of macrophages pretreated with SAR405 and GSK2795039, respectively (Figure 5E, 5F and 5G). Interestingly, inhibition of LAP significantly reduced the production of IL-10 upon *T. whipplei* infection (Figure 5H).

Altogether, these results suggest that LAP induction plays a dual role during *T. whipplei* infection. Indeed, we show that *T. whipplei* infection triggers an early blockade of autophagy which is further reinforced over time by the infection-induced overexpression of RUBCN. Additionally, we showed that LAP inhibition disrupts the anti-inflammatory IL-10 response, suggesting that *T. whipplei* uptake through LAP also plays a critical role in creating a microenvironment conducive to bacterial replication within macrophages.

## Discussion

The hallmark of Whipple’s disease is the infiltration of the lamina propria by periodic acid-Schiff-positive macrophages. Both *in vitro* and *in vivo*, the survival and replication of *T. whipplei* are associated with the induction of a M2 polarization profile characterized by the expression of anti-inflammatory cytokines leading to a Th2-skewed immune response [19,22,26]. In macrophages, *T. whipplei* interferes with phagosome maturation and replicates in a late phagosome lacking cathepsin D, suggesting a defect in phagosome-lysosome fusion [27]. However, the molecular pathways activated upon bacterial uptake remain relatively unknown.

In the present study, we showed that macrophages could internalize *T. whipplei* by LAP. Bacteria further escaped the LAPosome before being recaptured by xenophagy. This was supported by different imaging approaches showing bacteria colocalizing with cytosolic proteins and found in double-membrane compartments. Bacteria then sustainably blocked the autophagic flux and maturation of the autophagosome allowing the constitution of *T. whipplei* replicative niche. These events were probably reminiscent from LAP induction since inhibition of LAP restored the autophagic flux and drastically decreased IL-10 production.

LAP is characterized by the assistance of some components of the canonical autophagy machinery to phagosomal membranes. Although not fully understood, induction of the LAP pathway is mediated by the interaction of extracellular cargos with surface pattern recognition receptors including Toll-like receptors, C-type lectin receptors or Fcγ receptors [28–30]. We previously showed that host responses to *T. whipplei* were at least in part dependent on MyD88 and/or TRIF pathways [22]. Uptake of *T. whipplei* by LAP may therefore involve the Toll-like receptor recognition of a yet to define *T. whipplei* ligand. In addition, during LAP, the multimeric phosphotidylinositol 3-kinase complex (PI3KC3) also include UVRAG and RUBCN, the latter being essential for LAP maturation through the generation of PI3P and stabilization of the NOX2 complex at the phagosome membrane. This results in the generation of reactive oxygen species (ROS) whose production leads to the rapid lipidation of LC3 [31]. We found that after 2 hours of infection, most bacteria colocalized with RUBCN, UVRAG, NOX2 and LC3, which clearly suggests that bacterial uptake in macrophages involves LAP. This is further strengthened by the fact that inhibition of LAP by treating the cells with SAR405 which is a PIK3C3/Vps34 specific inhibitor [32] and with the NOX2 inhibitor GSK2795039 significantly reduced internalization of bacteria by macrophages. Involvement of LAP in the uptake of *M. marinum* and *M. tuberculosis*, which are somehow phylogenetic relatives of *T. whipplei* has previously been described in macrophages [9,33]. Interestingly, *M. marinum* and *M. tuberculosis* both escape macrophage bactericidal activity, but this involves different mechanisms. Indeed, *M. marinum* resides in a vacuole with features of a late acidic phagosomal compartment expressing Rab7, LAMP2, but devoid of the lysosomal protease cathepsin D, while *M. tuberculosis* evades LAP by excluding NOX2 from the vacuole [9,33]. In the case of *T. whipplei* the mechanism seems even more complex. Indeed, although it was shown that similar to *M. marinum, T. whipplei* replicates in a Rab7-, LAMP2-, v-ATPase-positive compartment which lacks cathepsin D [27], transmission electron microscopy analysis revealed free *T. whipplei* bacteria in the cytosol and the elongation of a double membrane structure typical of an autophagophore. The fact that *T. whipplei* could escape the phagosome was unexpected although the bacteria have previously been observed free in the cytosol of epithelial cells [34]. Translocation from the phagosome to the cytosol required bacterial viability, suggesting that this is an active process or at least a mechanism dependent on *de novo* protein synthesis. *M. tuberculosis* and *M. leprae* progressively translocate from phagolysosomes into the cytosol in nonapoptotic cells while *M. bovis* BCG or heat-killed mycobacteria do not [35]. In fact, this process depends on the secretion of CFP-10 and ESAT-6 whose genes are absent in *M. bovis* BCG [35]. To date, no such virulence factors have been described for *T. whipplei*, further studies are required for identifying candidates involved in *T. whipplei* translocation to the cytosol.

In line with bacterial escape in the cytosol, we found that *T. whipplei* colocalized with galectin 8 and NDP52. Galectin-8 has been reported to tag bacterial glycoproteins and, together with NDP52, is recruited when the integrity of phagocytic vacuoles is compromised [36]. Cells lacking galectin-8 fail in controlling *S. enterica* serovar Typhimurium replication [37]. Accumulation of galectin-8 on damaged vacuoles or bacteria allows the ligation of the cargo receptor NDP52 which then initiates phagophore formation by recruiting the ULK complex [38]. Interestingly, we found that 40% of bacteria colocalized with ULK1 at 2 h while 70 to 75% colocalized with galectin 8 and NDP52, suggesting that the ULK1 complex leaves the omegasome before the complete closure of the xenophagy-derived vacuole, as previously observed [39]. Accordingly, after 24 hours *T. whipplei* was found in LC3-positive autophagic vacuoles expressing ATG5-12 and BECN1 which also harbored mTOR and Raptor proteins as previously described on the membrane of the *Salmonella* vacuole [40]. Interestingly and in agreement with previously published data [17], we found that *T. whipplei* did not colocalize with the receptor protein SQSTM1/p62 and was not systematically ubiquitinated although ubiquitin accumulated in infected cells. This is in sharp contrast with observations made with *S. enterica* Typhimurium *s*howing that SQSTM1/p62 was recruited to *S. enterica* Typhimurium targeted by autophagy, and that this recruitment was associated with accumulation of ubiquitinated proteins localized to the bacteria and the restriction of intracellular replication [41]. The lack of colocalization between *T. whipplei* and members of the ESCRT machinery, including TSG101, CHMP4A and VPS4 further strengthens our findings since ESCRT complexes recognize and cluster ubiquitinated cargos via their ubiquitin-binding domains and direct them to endomembrane sites [23]. As we did not find ubiquitin colocalization with *T. whipplei*, it is not surprising that ESCRT members are not colocalizing with *T. whipplei*. Some bacteria, including *L. pneumophila* have been shown to manipulate host ubiquitin deposition by expressing deubiquitinating enzymes (DUBs), resulting in the exclusion of SQSTM1/p62 from the vacuole [42,43]. Whether a similar mechanism is used by *T. whipplei* remains to be determined, although no such enzymes have been detected in *T. whipplei* genome.

In addition, *T. whipplei*-containing autophagosomes lack UVRAG which was found at the periphery of the cells. UVRAG plays a critical role in the final step of auto/xenophagy since it is recruited to the BECN1/VPS34 complex to promote the fusion of autophagosome with lysosome [44]. Here, the exclusion of UVRAG from *T. whipplei*-containing autophagosomes may arise from the sustained over expression of RUBCN, both at the transcriptional and translational levels. Indeed, RUBCN interacts with UVRAG to inhibit fusion of autophagosomes and endosomes with lysosomes [45]. Hence, the fact that *T. whipplei* did not colocalize with SQSTM1/p62 and UVRAG may explain why bacteria escape killing and replicate in macrophages in a late acidic compartment [18]. Therefore, it is likely that *T. whipplei* replication initiates after that free bacteria have been recaptured by xenophagy since it was estimated that *T. whipplei* doubling time approximates 32 to 36 hours [46]. In addition, it was shown that *T. whipplei* replication required phagosome acidification since alkalinizing reagents, which increase the intravacuolar pH, promote the killing of *T. whipplei* in HeLa cells [15].

The defect of phagosome maturation and fusion with lysosome undoubtedly reflects a more general phenomenon of interference with the autophagy flux which conditions the outcome of intracellular pathogen survival [47]. It is therefore not surprising that we observed a blockade of the autophagic flux with an accumulation of SQSTM1/p62 and LC3-II cargoes in macrophages. This blockade was not compensated by activation of canonical autophagy with rapamycin or starvation and was reinforced by late autophagy inhibitors such as bafilomycin A1 or chloroquine. This suggests that *T. whipplei* blocks the autophagic flux at a late stage which cannot be compensated by autophagy inducers. Similar findings were obtained upon infection of macrophages with *Staphylococcus aureus* [48] or *Brucella suis* [49], indicating that blocking the autophagic flux at a late stage is a common strategy used by intracellular bacteria.

Finally, we found that if late-stage blockade of the autophagic flux seems to participate in the elaboration of *T. whipplei* replicating niche, early activation of LAP may interfere with macrophage activation and contribute to immunomodulation. Indeed, we previously showed that *T. whipplei* promotes M2 macrophage activation [19,22]. Interestingly, we found that LAP inhibition with SAR405 and GSK2795039 completely abolished IL-10 secretion without affecting that of TNF, IL-1β and IL-6 in *T. whipplei*-infected macrophages. Consistent with our observations, it was shown that LAP regulates the polarization of tumor-associated macrophages toward immunosuppressive functions by coordinating the metabolic reprogramming underlying macrophage polarization [25].

Finally, we showed that late-stage inhibition of autophagy with chloroquine resulted in a marked accumulation of LC3-II and SQSTM1/p62, suggesting that chloroquine may potentiate the blockade induced by *T. whipplei*. Of note, the chloroquine derivative hydroxychloroquine is, in combination with doxycycline, the recommended treatment for Whipple’s disease [50]. Indeed, as a weak base, chloroquine may alkalify the *T. whipplei*-containing vacuole, and restrict bacterial replication [15]. In addition, although hydroxychloroquine is also used in the treatment of systemic lupus erythematosus and rheumatoid arthritis for its immunomodulatory properties [51], it paradoxically reprograms tumor-associated macrophages to a M1 inflammatory profile [52] and restores MHC-I levels, leading to improved antigen presentation and enhanced anti-tumor responses [53,54]. Hence, while the *in vitro* benefits of autophagy inducers are being studied, notably for the treatment against mycobacteria [55], autophagy inhibitors with versatile effects such as chloroquine allow killing of *S. aureus* in murine macrophages [56]. Further studies are required to delineate the precise contribution of chloroquine in the treatment of Whipple’s disease.

**Figure 6.**
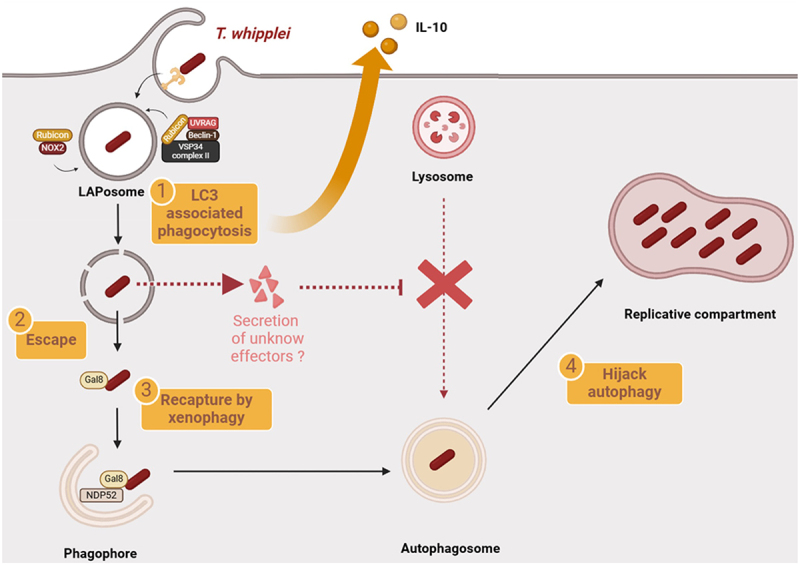
*T. whipplei* hijacks the autophagic flux. In macrophages, *T. whipplei* is enveloped in a LAPosome expressing RUBCN, and NOX2, characteristic of LC3-associated phagocytosis (1). LAP induction is associated with secretion of the immunomodulatory cytokine IL-10. *T. whipplei* then escapes (2) in the cytosol where it is tagged by Galectin 8 and NDP52 allowing the elongation of a phagophore (3). Subsequently, and probably thanks to the secretion of yet unknown bacterial factors, the bacterium hijacks the autophagic machinery (4), notably by blocking fusion and/or cargoes fusion with lysosomes, to invest its replicative niche which expresses LC3, Lamp1 but lacks cathepsin-D. Induction of LAP and perennial over-expression of RBCN could promote inhibition of autophagy and alternate activation of macrophage, by IL-10 secretion and favoring persistence of infection.

In conclusion, our results revealed a complex interplay between *T. whipplei* and autophagy-related pathways to create a replicative niche and promote immunomodulation (summarized in Figure 6). Such strategy probably involves virulence factors that are yet to identify and that could pave the way for targeted therapies of *T. whipplei* infections.

## Materials and Methods

### Isolation and culture of monocyte-derived macrophages (MDMs)

Peripheral blood mononuclear cells (PBMCs) were isolated by ficoll density gradient from buffy coats from donors obtained at the French Blood Bank after informed and consent of the donors according to the convention n°7828 established with the “Etablissement Français du Sang” (Marseille, France). As Whipple’s disease mainly affects males [57] and a sex bias has been described in autophagy-mediated diseases [58], all experiments were performed with cells from male donors. Monocytes were selected by 2 hours of adherence and differentiated into monocyte-derived macrophages (MDM) as previously described [20]. Briefly, monocytes were incubated in RPMI 1640 (Gibco, Thermo Fisher Scientific, 52400025) containing 10% human AB serum (MP Biomedicals, Thermo Fisher Scientific, 2938249) for 4 days, then in RPMI 1640 containing 10% Fetal Bovine Serum (FBS, Gibco, Thermo Fisher Scientific, A5256801), for 3 additional days.

### Bacteria and infection

MDMs were infected with *T. whipplei* strain Twist-Marseille (CNCM I-2202), cultured as previously described [59] with a bacterium to cell ratio of 50:1. When needed, gentamycin (50 µg/mL) was then added for 2 hours to ensure that no live bacteria were further internalized. In some experiments, bacteria were inactivated by 4% paraformaldehyde for 1 hour at room temperature, then washed twice and resuspended in Phosphate-buffered saline (PBS). Cells were treated or not with SAR405 (Sigma-Aldrich, 5330630001), GSK2795039 (Sigma-Aldrich, SML2770), Rapamycin (Sigma-Aldrich, R8781), Bafilomycin A1 (Sigma, B 1793) or chloroquine (Sigma, C6628) at the indicated timepoints. For starvation, cells were cultivated in RPMI 1640 without FBS 16 hours before infection.

### *DNA extraction and qPCR for internalization of* T. whipplei

MDMs were cultivated (5×10^5^ cells/well) in 24-well plates and treated or not 4 hours before infection. At the indicated timepoints, cells were washed with cold PBS, and lyzed with 1% Triton X-100. DNA was extracted using the EZNA Tissue DNA Kit (Omega, D3396-02 E.Z.N.A) and qPCR was performed using the SyberGreen Fast Master Mix (Roche Diagnostics, 03515869001) on a CFX96 Touch Real-Time PCR Detection System (Bio-Rad) with primers specific for *T. whipplei* in Table 1. For each qPCR run, a standard curve was generated using a serial dilution ranging from 10^9^ to 10^2^ copies of *T. whipplei* DNA.

**Table 1.** Primers used in this study.

	Target gene	Forward primer (5’-3’)	Reverse primer (5’-3’)
Beta actin	*ACTB*	GGAAATCGTGCGTGACATTA	AGGAAGGAAGGCTGGAAGAG
RUBCN	*RUBCN*	GTGATTCGGCACAGCTCTCT	ACTGGGAGGAAACGAAGGAT
*T. whipplei*	*leuS*	AAGAGCGAACCAGCCTTATG	CCTGTTCTATTGCCGGATTC

### RNA extraction and qRT-PCR

MDMs (5×10^5^ cells/well) were cultivated in 24 wells plates, infected for 4 hours, washed to remove free bacteria, and incubated for 12 days in RPMI 1640 containing 10% FBS. Every 3 days, cells were collected, and RNA was extracted using the RNeasy kit (Qiagen, 74106), treated with DNase I (Qiagen, 79254) before quantification with the NanoDrop Spectrophotometer (NanoDrop Technologies). RNA was retrotranscribed to cDNA using the Moloney Murine Leukemia Virus Reverse Transcriptase (MMLV-RT) kit (Invitrogen, 28025013) and oligo(dT) primers (Invitrogen, 18418020) before qPCR using the SyberGreen Fast Master Mix (Roche Diagnostics, 03515869001) on a CFX96 Touch Real-Time PCR Detection System (Bio-Rad) with the primers listed in Table 1. Expression of target genes was estimated based on the endogenous household beta actin (*ACTB*) gene and expressed as fold change (FC) using the following formula: FC = 2^−ΔΔCt^, where ΔΔCt = (Ct*_Target_* – Ct*_ACTB_*)_stimulated_ – (Ct*_Target_* – Ct*_ACTB_*)_unstimulated_.

### Immunofluorescence staining, confocal microscopy and colocalization analysis

MDMs (5×10^5^ cells/well), cultivated on glass coverslips were fixed with 4% paraformaldehyde for 15 minutes and permeabilized with 0.1% Triton X-100 in PBS for 3 minutes. Cells were saturated with 10% FBS in PBS for 1 hour at room temperature, then incubated with primary antibodies for 1 hour, washed with PBS, and incubated with secondary antibodies and 4′,6-diamidino-2-phenylindole (DAPI, Thermo Fisher Scientific, 10374168) for 1 hour. Antibodies used in this study are listed in Table 2. Coverslips were then washed, mounted with Mowiol and observed using LSM800 Airyscan confocal microscope (Zeiss) and a 63x oil objective. The degree of colocalization was expressed as Manders coefficient and calculated with the Zeiss ZEN software on at least 100 cells delimited by ROI and based on the integrated density of each signal independently for each different channel. Average fluorescence pixel intensity was measured with the Zeiss ZEN software according to a protocol adapted from [60]

**Table 2.** Antibodies used in this study.

Antibody	Provider	Dilution
*Immunofluorescence*		
Mouse anti-ATG5-12	Santa Cruz, sc-133158	1:250
Mouse anti-BECN1	Santa Cruz, sc-48341	1:250
Mouse anti-Galectin-8	Santa Cruz, sc-377133	1:200
Mouse anti-LC3B	Abcam, ab243506	1:250
Mouse anti-NDP52 or CALCOCO2	Santa Cruz, sc-376540	1:100
Mouse anti-NOX2 gp91-phox	Santa Cruz, sc-130543	1:200
Mouse anti-RUBCN	Sigma-Aldrich, AB4200838	1:250
Mouse anti-ULK1	Santa Cruz, sc-390904	1:250
Mouse anti-UVRAG	Santa Cruz, sc-293268	1:250
Mouse anti-Ubiquitin (P4D1)	Santa Cruz sc-8017	1:500
Mouse anti-p62 or SQSMT1	BD, 610833	1:500
Rabbit anti-Raptor	Abcam, ab26264	1:250
Rabbit anti-mTOR	Abcam, ab2732	1:250
Mouse anti-VPS4 (E-8)	sc-133122	1:250
Mouse anti-CHMP4A (E-6)	sc-514869	1:250
Mouse anti-TSG101 (C-2)	sc-7964	1:250
Serum from rabbit anti-*T. whipplei*	from [64]	1:1000
Rabbit anti-LC3B	Sigma, L7543	1:150
Goat polyclonal Anti-Mouse IgG (H+L) Alexa fluor 488	Thermo Fisher Scientific, A28175	1:250
Goat polyclonal Anti-Mouse IgG (H+L) Alexa fluor 555	Thermo Fisher Scientific, A32727	1: 250
Goat polyclonal Anti-Rabbit IgG (H+L) Alexa fluor 555	Thermo Fisher Scientific, A21428	1:250
Donkey polyclonal Anti-Rabbit IgG (H+L) Alexa fluor 488	Thermo Fisher Scientific, A21206	1: 250
*Western Blot*		
Rabbit anti-LC3B	Sigma, L7543	1:3000
Mouse anti-p62 or SQSMT1	BD, 610833	1:2000
Peroxidase-conjugated AffiniPure Donkey anti-rabbit IgG	Jackson Immuno Research, AB_10015282	1:5000
Peroxidase-conjugated AffiniPure Goat anti-mouse IgG	Jackson Immuno Research, AB_10015289	1:5000
Mouse anti-β actin peroxidase	Sigma, A3854	1:10 000

### Electron microscopy

MDMs were cultivated in modular well of removable single-break strip in a microplate (10^5^ cells/well), according to a protocol adapted from [61]. Substrate preparation was performed by Cell and Soft. Briefly, Greiner Bio-One 96 well single-break strip microplates (Greiner Bio-One, 705070) were UV-sterilized at 365 nm (125 mW/cm2) for 5 minutes (UV-KUB 1) under a laminar flow hood. Collagen coating was then performed under a sterile laminar flow cabinet. Subsequently, 400 ng of rat tail collagen I (Corning, 354236) was added to each well and incubated at 37°C overnight. Plates were stored at 4°C until use.

After infection, cells were fixed for at least 1 hour with 2.5% glutaraldehyde in 0.1 M sodium cacodylate buffer. Resin embedding was microwave-assisted with a PELCO BiowavePro+ (Ted Pella Inc.), by exchanging 200 µl of the different solutions at each step. Samples were washed two times with a mixture of 0.2 M saccharose/0.1 M sodium cacodylate and post-fixed with 1% OsO4 diluted in 0.2 M potassium hexa-cyanoferrate (III)/0.1 M sodium cacodylate buffer. After two washes with distilled water, samples were gradually dehydrated by successive baths in 30%, 50%, 70%, 90%, 96%, and 100% ethanol. Substitution with Epon resin (Embed 812 mixed with NMA, DDSA, and DMP-30 hardener; Electron Microscopy Sciences) was achieved by incubations with 25%, 50%, 75% Epon resin in ethanol and incubations with 100% Epon resin. Polymerization occurred with cells in 100% fresh Epon for 72 hours at 60°C. All solutions used above were 0.2 µm filtered. Resin blocks were placed in a UC7 ultramicrotome (Leica Biosystems), trimmed to pyramids, and ultrathin 70 nm sections were cut and placed on HR25 300 Mesh Copper/Rhodium grids (TAAB). Sections were contrasted according to Reynolds [62]. Electron micrographs were obtained on a Tecnai G2 TEM (Thermo-Fischer/FEI) operated at 200 keV equipped with a 4096 × 4096 pixels resolution Eagle camera (FEI).

### Western blot

MDMs (10^6^ cells/well) cultivated in 6-well plate were lyzed with RIPA buffer (20 nM Tris-HCl, 200 nM NaCl, 1 mm EDTA, 1% Triton X-100, pH 7.5). The protein content was determined by the Bradford method [63]. Equal amounts of protein (30 µg) were separated by 12% sodium dodecyl sulfate-polyacrylamide gel electrophoresis (SDS-PAGE), transferred to 0.45 µm nitrocellulose membrane (Bio Rad, 1620115), and analyzed by western blot. Membranes were saturated using 0.05% Tween 20 in PBS supplemented with 5% powdered milk for 1 hour and incubated with primary antibodies (Table 2) overnight at 4°C on a shaker. Membranes were then washed and incubated with corresponding horseradish peroxidase (HRP)-conjugated secondary antibody for 1 hour (Table 2). Proteins were visualized using chemiluminescence reaction HRP Substrate (Millipore), and image acquisition was done by using a Fusion Fx imaging system (Viller Lourmat). The bands were adjusted within the linear range and quantified using ImageJ/Fiji (National Institutes of Health) 1.53c.

### Immunoassays

Cytokine release was evaluated from supernatants of MDMs at 24 hours post-infection. Tumor necrosis factor (TNF, Invitrogen, KHC3011), Interleukin (IL)-1β/ IL-1F2 (R&D Systems, HSBB00D), IL-10 and IL-6 (BD OptEIA, 550613 and 550799) were quantified according to the manufacturer’s recommendations. The sensitivity (pg/ml) was 8, 0.125, 15.4 and 3.9 for TNF, IL-1β, IL-6 and IL-10, respectively.

### Statistical analysis

Statistical analysis was performed with GraphPad Prism 8.4.0 and statistical significance was considered for p values below 0.05. Details for individual analyses are provided in the figure legends. Unless otherwise noted, data are presented as mean ± standard deviation.

## Supplementary Material

Supplementary figures.docx

## Abbreviations

ATG: autophagy-related gene
IL: Interleukin
IFN: Interferon
CALCOCO2/NDP52: autophagy receptor calcium binding and coiled-coil domain 2
LAP: LC3-associated phagocytosis
LC3: Microtubule-associated proteins 1A/1B light chain 3B
MDM: Monocyte-derived macrophage
NADPH oxidase: nicotinamide adenine dinucleotide phosphate oxidase
NDP52: nuclear dot protein 52
NOX2: NADPH oxidase 2
ROS: Reactive Oxygen Species
RUBCN: RUN and cysteine rich domain containing BECN1-interacting protein
SQSTM1: sequestosome
TNF: Tumor Necrosis Factor
ULK1: unc-51-like autophagy activating kinase 1
UVRAG: UV radiation resistance associated.

## References

[1] Jang YJ, Kim JH, Byun S. Modulation of Autophagy for Controlling Immunity. Cells. 2019;8(2):138. 30744138 10.3390/cells8020138 PMC6406335

[2] Netea-Maier RT, Plantinga TS, van de Veerdonk FL, et al. Modulation of inflammation by autophagy: Consequences for human disease. Autophagy. 2016;12(2):245–24. 26222012 10.1080/15548627.2015.1071759 PMC4836004

[3] Tao S, Drexler I. Targeting Autophagy in Innate Immune Cells: Angel or Demon During Infection and Vaccination? Front Immunol. 2020;11:460. 32265919 10.3389/fimmu.2020.00460 PMC7096474

[4] Wong S-W, Sil P, Martinez J. Rubicon: LC3-associated phagocytosis and beyond. FEBS J. 2018;285(8):1379–1388. 29215797 10.1111/febs.14354 PMC6779045

[5] Gluschko A, Farid A, Herb M, et al. Macrophages target Listeria monocytogenes by two discrete non-canonical autophagy pathways. Autophagy. 2022;18(5):1090–1107. 34482812 10.1080/15548627.2021.1969765 PMC9196813

[6] Masud S, Prajsnar TK, Torraca V, et al. Macrophages target Salmonella by Lc3-associated phagocytosis in a systemic infection model. Autophagy. 2019;15(5):796–812. 30676840 10.1080/15548627.2019.1569297 PMC6526873

[7] Riebisch AK, Mühlen S, Beer YY, et al. Autophagy-A Story of Bacteria Interfering with the Host Cell Degradation Machinery. Pathogens. 2021;10(2):110. 33499114 10.3390/pathogens10020110 PMC7911818

[8] Siqueira M da S, Ribeiro R de M, Travassos LH. Autophagy and Its Interaction With Intracellular Bacterial Pathogens. Front Immunol. 2018;9:935. 29875765 10.3389/fimmu.2018.00935 PMC5974045

[9] Köster S, Upadhyay S, Chandra P, et al. Mycobacterium tuberculosis is protected from NADPH oxidase and LC3-associated phagocytosis by the LCP protein CpsA. Proc Natl Acad Sci U S A. 2017;114(41):E8711–E8720. 10.1073/pnas.1707792114 PMC5642705 28973896

[10] Anes E, Pires D, Mandal M, et al. ESAT-6 a Major Virulence Factor of Mycobacterium tuberculosis. Biomolecules. 2023;13(6):968. 37371548 10.3390/biom13060968 PMC10296275

[11] Augenstreich J, Briken V. Host Cell Targets of Released Lipid and Secreted Protein Effectors of Mycobacterium tuberculosis. Front Cell Infect Microbiol. 2020;10:595029. 33194845 10.3389/fcimb.2020.595029 PMC7644814

[12] Shariq M, Quadir N, Alam A, et al. The exploitation of host autophagy and ubiquitin machinery by Mycobacterium tuberculosis in shaping immune responses and host defense during infection. Autophagy. 2023;19(1):3–23. 35000542 10.1080/15548627.2021.2021495 PMC9809970

[13] Marth T, Moos V, Müller C, et al. Tropheryma whipplei infection and Whipple’s disease. Lancet Infect Dis. 2016;16(3):e13–22. 26856775 10.1016/S1473-3099(15)00537-X

[14] Desnues B, Ihrig M, Raoult D, et al. Whipple’s disease: a macrophage disease. Clin Vaccine Immunol. 2006;13(2):170–178. 16467322 10.1128/CVI.13.2.170-178.2006 PMC1391942

[15] Ghigo E, Capo C, Aurouze M, et al. Survival of Tropheryma whipplei, the agent of Whipple’s disease, requires phagosome acidification. Infect Immun. 2002;70(3):1501–1506. 11854238 10.1128/IAI.70.3.1501-1506.2002 PMC127739

[16] Mottola G. The complexity of Rab5 to Rab7 transition guarantees specificity of pathogen subversion mechanisms. Front Cell Infect Microbiol. 2014;4:180. 25566515 10.3389/fcimb.2014.00180 PMC4273659

[17] Desnues B, Raoult D, Mege J-L. IL-16 is critical for Tropheryma whipplei replication in Whipple’s disease. J Immunol. 2005;175(7):4575–4582. 16177102 10.4049/jimmunol.175.7.4575

[18] Ghigo E, Barry AO, Pretat L, et al. IL-16 promotes T. whipplei replication by inhibiting phagosome conversion and modulating macrophage activation. PLOS ONE. 2010;5(10):e13561. 10.1371/journal.pone.0013561 PMC2958842 21042409

[19] Desnues B, Lepidi H, Raoult D, et al. Whipple disease: intestinal infiltrating cells exhibit a transcriptional pattern of M2/alternatively activated macrophages. J Infect Dis. 2005;192(9):1642–1646. 16206080 10.1086/491745

[20] Ben Azzouz E, Boumaza A, Mezouar S, et al. Tropheryma whipplei Increases Expression of Human Leukocyte Antigen-G on Monocytes to Reduce Tumor Necrosis Factor and Promote Bacterial Replication. Gastroenterology. 2018;155(5):1553–1563. 30076840 10.1053/j.gastro.2018.07.034

[21] Gorvel L, Al Moussawi K, Ghigo E, et al. Tropheryma whipplei, the Whipple’s disease bacillus, induces macrophage apoptosis through the extrinsic pathway. Cell Death Dis. 2010;1(4):e34. 10.1038/cddis.2010.11 PMC3032299 21364641

[22] Al Moussawi K, Ghigo E, Kalinke U, et al. Type I interferon induction is detrimental during infection with the Whipple’s disease bacterium, Tropheryma whipplei. PLOS Pathog. 2010;6(1):e1000722. 10.1371/journal.ppat.1000722 PMC2798751 20090833

[23] Korbei B. Ubiquitination of the ubiquitin-binding machinery: how early ESCRT components are controlled. Essays Biochem. 2022;66(2):169–177. 35352804 10.1042/EBC20210042 PMC9400068

[24] Munafó DB, Colombo MI. A novel assay to study autophagy: regulation of autophagosome vacuole size by amino acid deprivation. J Cell Sci. 2001;114(Pt 20):3619–3629. 10.1242/jcs.114.20.3619 11707514

[25] Cunha LD, Yang M, Carter R, et al. LC3-Associated Phagocytosis in Myeloid Cells Promotes Tumor Immune Tolerance. Cell. 2018;175(2):429–441.e16. 30245008 10.1016/j.cell.2018.08.061 PMC6201245

[26] Marth T, Kleen N, Stallmach A, et al. Dysregulated peripheral and mucosal Th1/Th2 response in Whipple’s disease. Gastroenterology. 2002;123(5):1468–1477. 12404221 10.1053/gast.2002.36583

[27] Mottola G, Boucherit N, Trouplin V, et al. Tropheryma whipplei, the agent of Whipple’s disease, affects the early to late phagosome transition and survives in a Rab5- and Rab7-positive compartment. PLoS One. 2014;9(2):e89367. 10.1371/journal.pone.0089367 PMC3933534 24586722

[28] Sanjuan MA, Dillon CP, Tait SWG, et al. Toll-like receptor signalling in macrophages links the autophagy pathway to phagocytosis. Nature. 2007;450 (7173):1253–1257. 18097414 10.1038/nature06421

[29] Tam JM, Mansour MK, Khan NS, et al. Dectin-1-dependent LC3 recruitment to phagosomes enhances fungicidal activity in macrophages. J Infect Dis. 2014;210(11):1844–1854. 24842831 10.1093/infdis/jiu290 PMC4271056

[30] Henault J, Martinez J, Riggs JM, et al. Noncanonical autophagy is required for type I interferon secretion in response to DNA-immune complexes. Immunity. 2012;37(6):986–997. 23219390 10.1016/j.immuni.2012.09.014 PMC3786711

[31] Heckmann BL, Green DR. LC3-associated phagocytosis at a glance. J Cell Sci. 2019;132(5):jcs222984. 10.1242/jcs.222984 PMC6432721 30787029

[32] Stempels FC, Janssens MH, Ter Beest M, et al. Novel and conventional inhibitors of canonical autophagy differently affect LC3-associated phagocytosis. FEBS Lett. 2022;596(4):491–509. 35007347 10.1002/1873-3468.14280

[33] Lerena MC, Colombo MI. Mycobacterium marinum induces a marked LC3 recruitment to its containing phagosome that depends on a functional ESX-1 secretion system. Cell Microbiol. 2011;13(6):814–835. 21447143 10.1111/j.1462-5822.2011.01581.x

[34] Friebel J, Schinnerling K, Weigt K, et al. Uptake of Tropheryma whipplei by Intestinal Epithelia. Int J Mol Sci. 2023;24(7):6197. 37047170 10.3390/ijms24076197 PMC10094206

[35] van der Wel N, Hava D, Houben D, et al. M. tuberculosis and M. leprae translocate from the phagolysosome to the cytosol in myeloid cells. Cell. 2007;129(7):1287–1298. 17604718 10.1016/j.cell.2007.05.059

[36] Bell SL, Lopez KL, Cox JS, et al. Galectin-8 Senses Phagosomal Damage and Recruits Selective Autophagy Adapter TAX1BP1 To Control Mycobacterium tuberculosis Infection in Macrophages. mBio. 2021;12(4):e0187120. 10.1128/mBio.01871-20 PMC8406326 34225486

[37] Thurston TLM, Wandel MP, von Muhlinen N, et al. Galectin 8 targets damaged vesicles for autophagy to defend cells against bacterial invasion. Nature. 2012;482 (7385): 414–418. 22246324 10.1038/nature10744 PMC3343631

[38] Ravenhill BJ, Boyle KB, von Muhlinen N, et al. The Cargo Receptor NDP52 Initiates Selective Autophagy by Recruiting the ULK Complex to Cytosol-Invading Bacteria. Mol Cell. 2019;74(2):320–329.e6. 30853402 10.1016/j.molcel.2019.01.041 PMC6477152

[39] Karanasios E, Stapleton E, Manifava M, et al. Dynamic association of the ULK1 complex with omegasomes during autophagy induction. J Cell Sci. 2013;126(Pt 22):5224–5238. 10.1242/jcs.132415 24013547

[40] Tattoli I, Sorbara MT, Vuckovic D, et al. Amino acid starvation induced by invasive bacterial pathogens triggers an innate host defense program. Cell Host Microbe. 2012;11(6):563–575. 22704617 10.1016/j.chom.2012.04.012

[41] Zheng YT, Shahnazari S, Brech A, et al. The adaptor protein p62/SQSTM1 targets invading bacteria to the autophagy pathway. J Immunol. 2009;183(9):5909–5916. 19812211 10.4049/jimmunol.0900441

[42] Ge J, Wang Y, Chen X, et al. Phosphoribosyl-linked serine ubiquitination of USP14 by the SidE family effectors of Legionella excludes p62 from the bacterial phagosome. Cell Reports. 2023;42(8):112817. 37471226 10.1016/j.celrep.2023.112817

[43] Wehrmann M, Vilchez D. The emerging role and therapeutic implications of bacterial and parasitic deubiquitinating enzymes. Front Immunol. 2023;14:1303072. 38077335 10.3389/fimmu.2023.1303072 PMC10703165

[44] Paulus GLC, Xavier RJ. Autophagy and checkpoints for intracellular pathogen defense. Curr Opin Gastroenterol. 2015;31(1):14–23. 25394238 10.1097/MOG.0000000000000134 PMC4330559

[45] Sun Q, Westphal W, Wong KN, et al. Rubicon controls endosome maturation as a Rab7 effector. Proc Natl Acad Sci U S A. 2010;107(45):19338–19343. 20974968 10.1073/pnas.1010554107 PMC2984168

[46] Masselot F, Boulos A, Maurin M, et al. Molecular evaluation of antibiotic susceptibility: Tropheryma whipplei paradigm. Antimicrob Agents Chemother. 2003;47(5):1658–1664. 12709337 10.1128/AAC.47.5.1658-1664.2003 PMC153328

[47] Zhao YG, Codogno P, Zhang H. Machinery, regulation and pathophysiological implications of autophagosome maturation. Nat Rev Mol Cell Biol. 2021;22(11):733–750. 34302147 10.1038/s41580-021-00392-4 PMC8300085

[48] Cai J, Li J, Zhou Y, et al. Staphylococcus aureus facilitates its survival in bovine macrophages by blocking autophagic flux. J Cell Mol Med. 2020;24(6):3460–3468. 31997584 10.1111/jcmm.15027 PMC7131951

[49] Li J, Qi L, Diao Z, et al. Brucella BtpB Manipulates Apoptosis and Autophagic Flux in RAW264.7 Cells. Int J Mol Sci. 2022;23(22):14439. 36430916 10.3390/ijms232214439 PMC9693124

[50] Lagier J-C, Fenollar F, Lepidi H, et al. Treatment of classic Whipple’s disease: from in vitro results to clinical outcome. J Antimicrob Chemother. 2014;69(1):219–227. 23946319 10.1093/jac/dkt310

[51] Rainsford KD, Parke AL, Clifford-Rashotte M, et al. Therapy and pharmacological properties of hydroxychloroquine and chloroquine in treatment of systemic lupus erythematosus, rheumatoid arthritis and related diseases. Inflammopharmacology. 2015;23(5):231–269. 26246395 10.1007/s10787-015-0239-y

[52] Chen D, Xie J, Fiskesund R, et al. Chloroquine modulates antitumor immune response by resetting tumor-associated macrophages toward M1 phenotype. Nat Commun. 2018;9(1):873. 29491374 10.1038/s41467-018-03225-9 PMC5830447

[53] Yamamoto K, Venida A, Yano J, et al. Autophagy promotes immune evasion of pancreatic cancer by degrading MHC-I. Nature. 2020;581(7806):100–105. 10.1038/s41586-020-2229-5 PMC7296553 32376951

[54] Kimura T, Takabatake Y, Takahashi A, et al. Chloroquine in cancer therapy: a double-edged sword of autophagy. Cancer Res. 2013;73(1):3–7. 23288916 10.1158/0008-5472.CAN-12-2464

[55] Strong EJ, Lee S. Targeting Autophagy as a Strategy for Developing New Vaccines and Host-Directed Therapeutics Against Mycobacteria. Front Microbiol. 2020;11:614313. 33519771 10.3389/fmicb.2020.614313 PMC7840607

[56] Dey S, Bishayi B. Killing of Staphylococcus aureus in murine macrophages by chloroquine used alone and in combination with ciprofloxacin or azithromycin. J Inflamm Res. 2015;8:29–47. 25653549 10.2147/JIR.S76045 PMC4309780

[57] Boumaza A, Ben Azzouz E, Arrindell J, et al. Whipple’s disease and Tropheryma whipplei infections: from bench to bedside. Lancet Infect Dis. 2022;22(10):e280–e291. 35427488 10.1016/S1473-3099(22)00128-1

[58] Shang D, Wang L, Klionsky DJ, et al. Sex differences in autophagy-mediated diseases: toward precision medicine. Autophagy. 2021;17(5):1065–1076. 32264724 10.1080/15548627.2020.1752511 PMC8143224

[59] Renesto P, Crapoulet N, Ogata H, et al. Genome-based design of a cell-free culture medium for Tropheryma whipplei. Lancet. 2003;362(9382):447–449. 10.1016/S0140-6736(03)14071-8 12927433

[60] Palikaras K, SenGupta T, Nilsen H, et al. Assessment of dopaminergic neuron degeneration in a C. elegans model of Parkinson’s disease. STAR Protoc. 2022;3(2):101264. 35403008 10.1016/j.xpro.2022.101264 PMC8983426

[61] Le Bideau M, Wurtz N, Baudoin J-P, et al. Innovative Approach to Fast Electron Microscopy Using the Example of a Culture of Virus-Infected Cells: An Application to SARS-CoV-2. Microorganisms. 2021;9(6):1194. 34073053 10.3390/microorganisms9061194 PMC8228702

[62] Reynolds ES. The use of lead citrate at high pH as an electron-opaque stain in electron microscopy. J Cell Biol. 1963;17(1):208–212. 13986422 10.1083/jcb.17.1.208 PMC2106263

[63] Kruger NJ. The Bradford method for protein quantitation. Methods Mol Biol. 1994;32:9–15. 7951753 10.1385/0-89603-268-X:9

[64] La Scola B, Fenollar F, Fournier PE, et al. Description of Tropheryma whipplei gen. nov. sp. nov. the Whipple’s disease bacillus. Int J Syst Evol Microbiol. 2001;51(Pt 4):1471–1479. 10.1099/00207713-51-4-1471 11491348

