# Supplementary material for: Tropheryma whipplei escapes LAPosome and modulates macrophage response in a xenophagy-dependent manner
Source: Autophagy Rep. 2025 Mar 11;4(1):2475527. doi: 10.1080/27694127.2025.2475527 (PMC11921966; doi:10.1080/27694127.2025.2475527)
Supplement: Supplementary figures.docx [file KAUO_A_2475527_SM9837.docx]

**
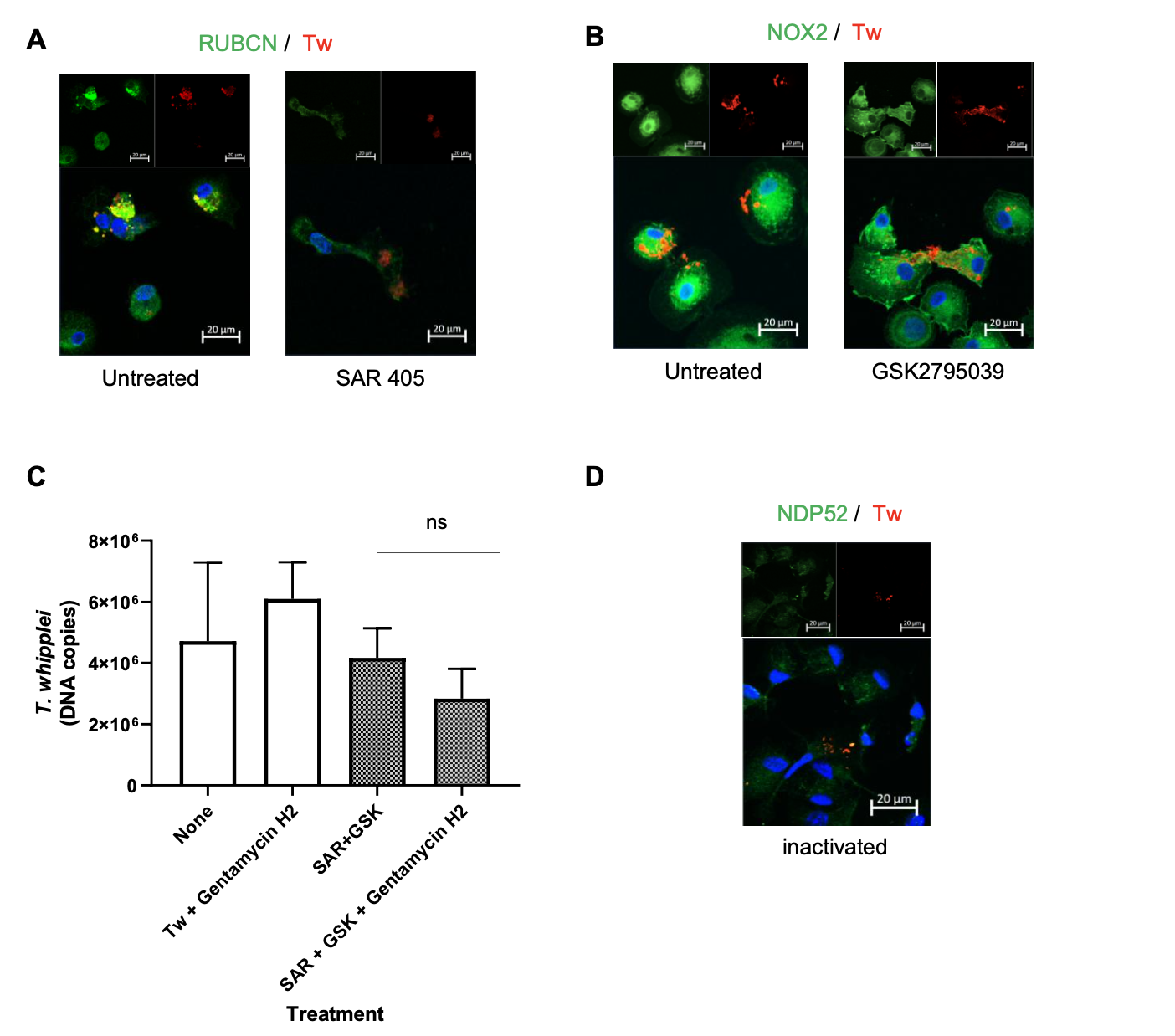
**

**Figure S1. LAP inhibition.** MDMs were pre-treated or not with 10 µM of SAR405, or 25 µM of GSK2795039 for 4 hours before been infected with *T. whipplei* (50 bacteria per cell) for 2 h. Cells were then fixed, and stained with anti-*T. whipplei* antibody in red, anti-RUBCN (A) or NOX2 (B). Nuclei were stained with DAPI (blue). The images were visualized by confocal fluorescence microscopy. Scale bar: 20 µm. The experiments were performed in three different donors (N = 3); representative results are shown. (C) MDMs were pre-treated with 10 µM of SAR405 and 25 µM of GSK2795039 for 4 hours before been infected with *T. whipplei* (50 bacteria per cell) for 2 h. Cells were then washed, treated during 2 additional hours with 50 µg/mL gentamycin in fresh medium before washing and then lysed. Bacterial DNA copies was determined by qPCR. The experiment was performed using three different donors (N = 3), and the values represent the mean ± standard error of the mean. *p<0.05, by two-way ANOVA. (D) MDMs infected with PFA-inactivated *T. whipplei* (50 bacteria per cell) for 2 h. Cells were then fixed and stained with anti-*T. whipplei* antibody in red and NDP52 in green. Nuclei were stained with DAPI (blue). The images were visualized by confocal fluorescence microscopy. Scale bar: 20 µm. The experiments were performed in three different donors (N = 3); representative results are shown.


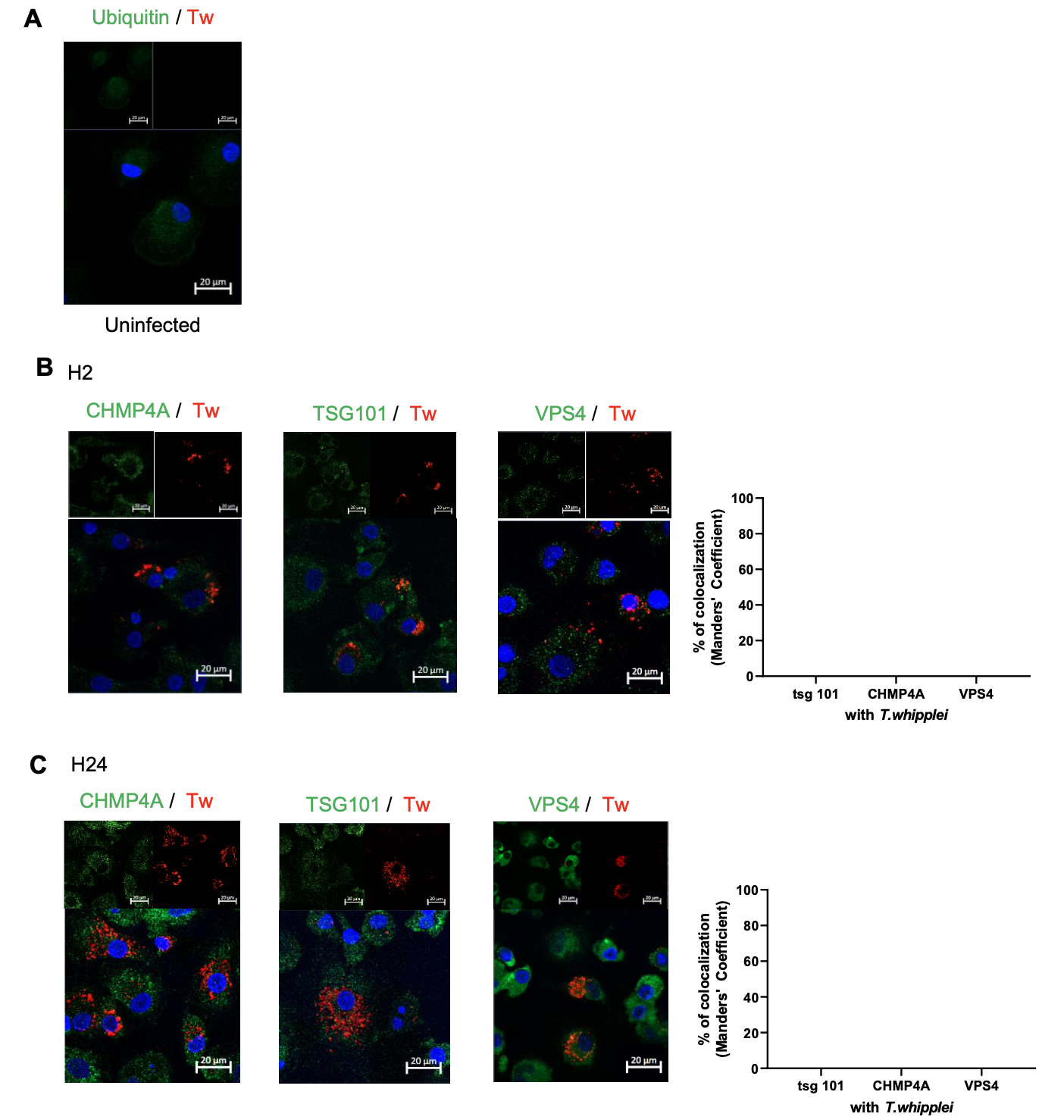


**Figure S2. Ubiquitin-dependent xenophagy and ESCRT-system failed to catch *T. whipplei*.** (A) MDMs were left uninfected and then fixed after 2-hour incubation and stained with an anti-*T. whipplei* antibody in red and antibodies directed against Ubiquitin in green. Nuclei were stained with DAPI (blue). (B-C) MDMs were infected with *T. whipplei* (50 bacteria per cell) for 2 (B) or 24 hours (C), fixed, then stained with an anti-*T. whipplei* antibody in red and antibodies directed against ESCRT-system effectors CHMP4A, TSG101 or VPS4 in green. Nuclei were stained with DAPI (blue). The images were visualized by confocal fluorescence microscopy. Scale bar: 20 µm. Colocalization between *T. whipplei* and the proteins of interest was expressed as Manders coefficient. The experiments were performed in triplicates (N = 3); representative results are shown.


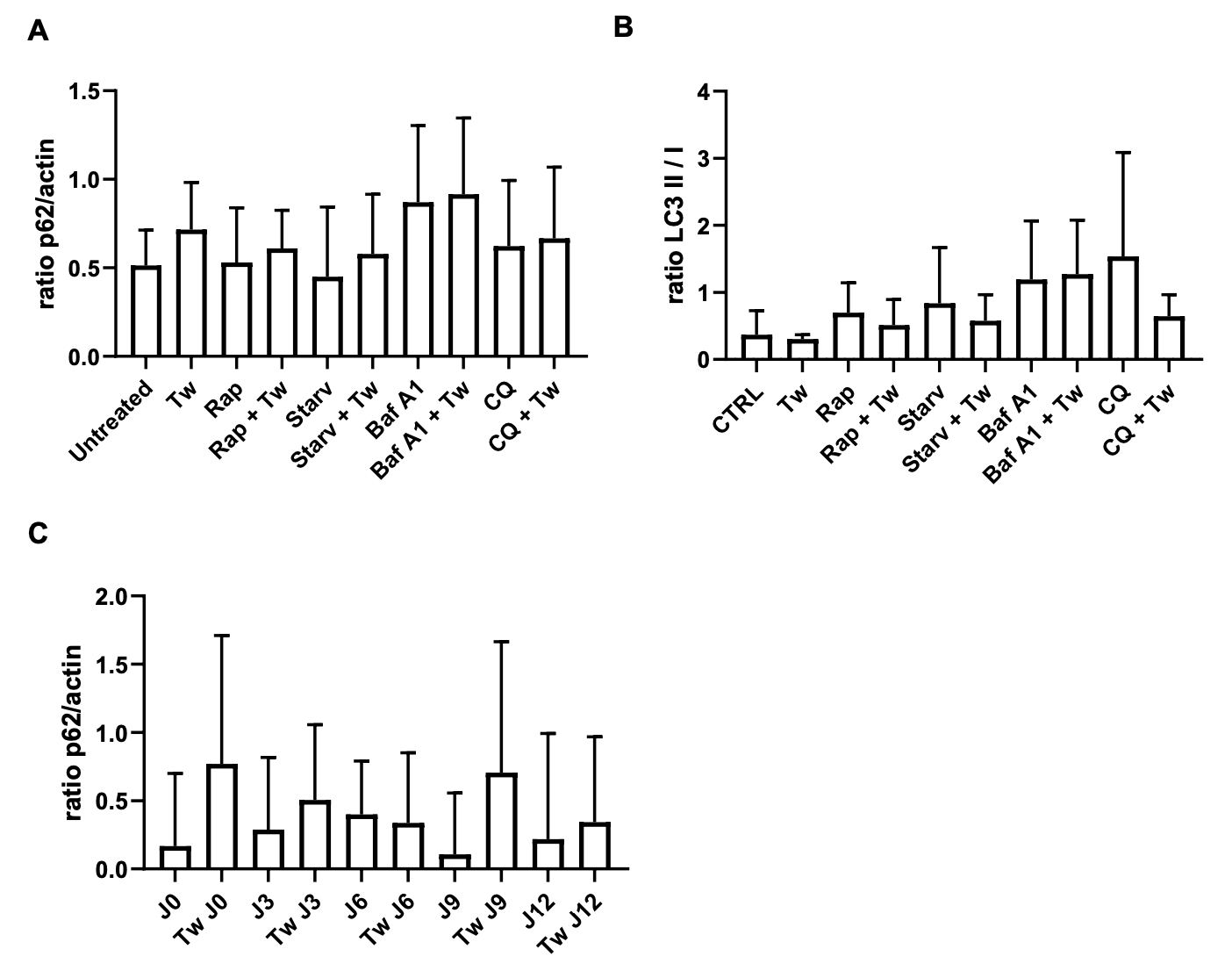


**Figure S3. *T. whipplei* hijacks the autophagic flux in macrophages.** MDMs from three individuals were pre-treated or not for 4 hours with 500 nM rapamycin, or starved for 16 hours, or treated with 200 nM bafilomycin A1, or 60 µM chloroquine, before infection with *T. whipplei* (50 bacteria per cell) for 24 hours. Cells were then washed, lysed and whole-cell lysates were analyzed by western blot. The ratio between p62 and actin (A) or LC3-II and LC3-I (B) were calculated after quantification by densitometry. (C) MDMs were infected with *T. whipplei* (50 bacteria per cell) for 4 hours, washed to remove free bacteria and incubated for 12 days (0 corresponds to 4h infection). Every 3 days, cells were washed and lysed in RIPA buffer. Whole-cell lysates were then analyzed by western blot against the indicated proteins. The ratios between p62 and actin were calculated after quantification by densitometry. The experiments were performed in three different donors, bars represent the mean ± standard error (N = 3).
